# Spatial epidemiology of skin cancer in Iran: separating sun-exposed and non-sun-exposed parts of the body

**DOI:** 10.1186/s13690-022-00798-2

**Published:** 2022-01-20

**Authors:** Behzad Kiani, Parinaz Tabari, Alireza Mohammadi, Sayyed Mostafa Mostafavi, Mohsen Moghadami, Mitra Amini, Abbas Rezaianzadeh

**Affiliations:** 1 Department of Medical Informatics, School of Medicine, Mashhad University of Medical Sciences, Mashhad, Iran; 2 Clinical Education Research Center, Shiraz University of Medical Sciences, Shiraz, Iran; 3 School of Allied Medical Sciences, Tehran University of Medical Sciences, Tehran, Iran; 4 Department of Geography and Urban Planning, Faculty of Social Sciences, University of Mohaghegh Ardabili, Ardabil, Iran; 5 Non-Communicable Diseases Research Center, Shiraz University of Medical Sciences, Shiraz, Iran; 6 Colorectal Research Center, Shiraz University of Medical Sciences, Shiraz, Iran

**Keywords:** Skin cancer, Spatial analysis, Spatial autocorrelation, Cluster analysis, Geographical information systems, Iran, Sun exposure

## Abstract

**Background:**

Skin cancer is among the most common cancer types with an increasing global trend of incidence rate. This study explores the spatial distribution of skin cancer, considering body sites exposed and not exposed to sunshine separately.

**Methods:**

We used 4302 skin cancer cases recorded by Fars Cancer Registry in south-western Iran for over 6 years (2011–2017). The variables included in the study were patients’ residence address, gender, age, report date, and final topographical code. The patients’ addresses were geocoded to the counties of the study area. Skin cancer sites were categorized based on sun exposure in male and female cases. We used the empirical Bayesian smoothing approach to smooth the skin cancer incidence rate at the county level to remove any potential population size bias. Finally, Anselin’s Local Moran’s Index and Getis Ord G* were used to identify the clustered and high-risk skin cancer geographical areas.

**Results:**

The incidence rates had an increasing trend from 14.28 per 100,000 people in 2011 to 17.87 per 100,000 people in 2016, however, it was decreased to 13.05 per 100,000 people in 2017. Out of 4302 patients with skin cancer, 2602 cases (60%) were male. The cancer cumulative incidence rate in males and females who were not exposed to sunshine was 7.80 and 14.18 per 100,000, respectively. The rates increased to 86.22 and 48.20 in males and females who were exposed to the sun. There were some high-risk spatial clusters of skin cancer in the study area. Further investigations are required to identify the underlying cause of the formation of these clusters.

**Conclusions:**

Patients exposed to sunshine, especially among the male group, experienced much higher rates of cancer occurrence as compared to unexposed individuals. With a heterogeneous spatial pattern, hotspots were identified in non-sun-exposed and sun-exposed categories in the study area. Researchers and policymakers can significantly benefit from the spatial analyses of skin cancer incidence. These analyses can provide useful and timely prevention policies as well as tailored monitoring techniques in high-risk regions.

**Supplementary Information:**

The online version contains supplementary material available at 10.1186/s13690-022-00798-2.

## Background

Skin cancer is among the most common categories of cancer with an increasing global trend of occurrence [1]. Malignant melanoma (MM) and Non-Melanoma Skin Cancer (NMSC) are the two main skin cancer types. NMSCs are categorized as Basal Cell Carcinoma (BCC), Squamous Cell Carcinoma (SCC), and Bowen’s disease. The incidence of NMSC and MM is on a growing trend [2]. In Iran, it is also reported that skin cancer is more prevalent compared to other types of cancer, and BCC is the most common morphologic form of skin cancer in this country [3].

Melanoma, SCC, and BCC manifestations have been correlated with diverse geographic locations as well as the amount of ultraviolet (UV) radiation [4]. The effect of outdoor activities, environmental pollution, and radiation exposure on skin cancer occurrence have been previously documented in the literature [5]. The elevated prevalence in the male gender accounts for higher sunshine exposure without suitable protection during outside activities. In numerous occupational circumstances, individuals are unprotected when exposed to UV radiation [6]. Long term exposure to UV radiation in sunlight is well recognized to be responsible for most NMSCs in humans [7].

Conducting a spatial methodology using Geographical Information System (GIS) allows defining region-based patterns and trends associated with health outcomes, risk factors, and community wellbeing [8]. GIS is a computerized framework for storing, preserving, processing and presenting geographic information [9]. A study in Italy using Bayesian hierarchical spatial models highlighted that the risk of the cancers of melanoma and lip was incredibly significant for the coastal region and for body sites like the face and neck that are typically linked to high accumulated exposures, particularly in the male gender [10]. Studies in Brazil using GIS identified some spatial clusters with an elevated incidence and death rates of cutaneous melanoma [11, 12]. A study in Florida identified spatial clusters of high melanoma incidence rates and established a model to predict cases in neighborhoods with an increased risk of incidence [13]. BCC spatial clusters using spatial scan statistics were identified in northern California [14]. Satellite imagery and remote sensing were utilized to examine the relationship between the intensity of UV radiation and skin cancer in sexual and ethnic groups in the United States. The study revealed that men were more exposed to the radiation and more likely to develop skin cancer and the intensity of the radiation caused noticeable regional differences in different geographical areas in terms of skin cancer incidence [15]. In Iran, however, only a handful of relevant investigations have been undertaken regarding the geographic analysis of skin cancer. The high-risk regions of skin cancer at the province level were identified in Iran in 2015 and 2016. The studies investigated the relative risk of mortality and morbidity by skin cancer through spatial analysis [5, 16]. Another study in 2015 utilized GIS to investigate the geographic distribution of BCC in Ardabil Province, located in north-western Iran [17].

Based on a study in 2015, Fars Province had the highest incidence rate of skin cancer in southern Iran [18]. Therefore, the present study aims to conduct a geospatial analysis of skin cancer at the county level in the province of Fars, Iran. To the best of our knowledge, there is no study at the county level in Iran. Furthermore, the present study considers sunshine exposure to different skin sites in each gender, which has not been addressed before in Iran.

## Methods

### Study area

**Fig. 1:**
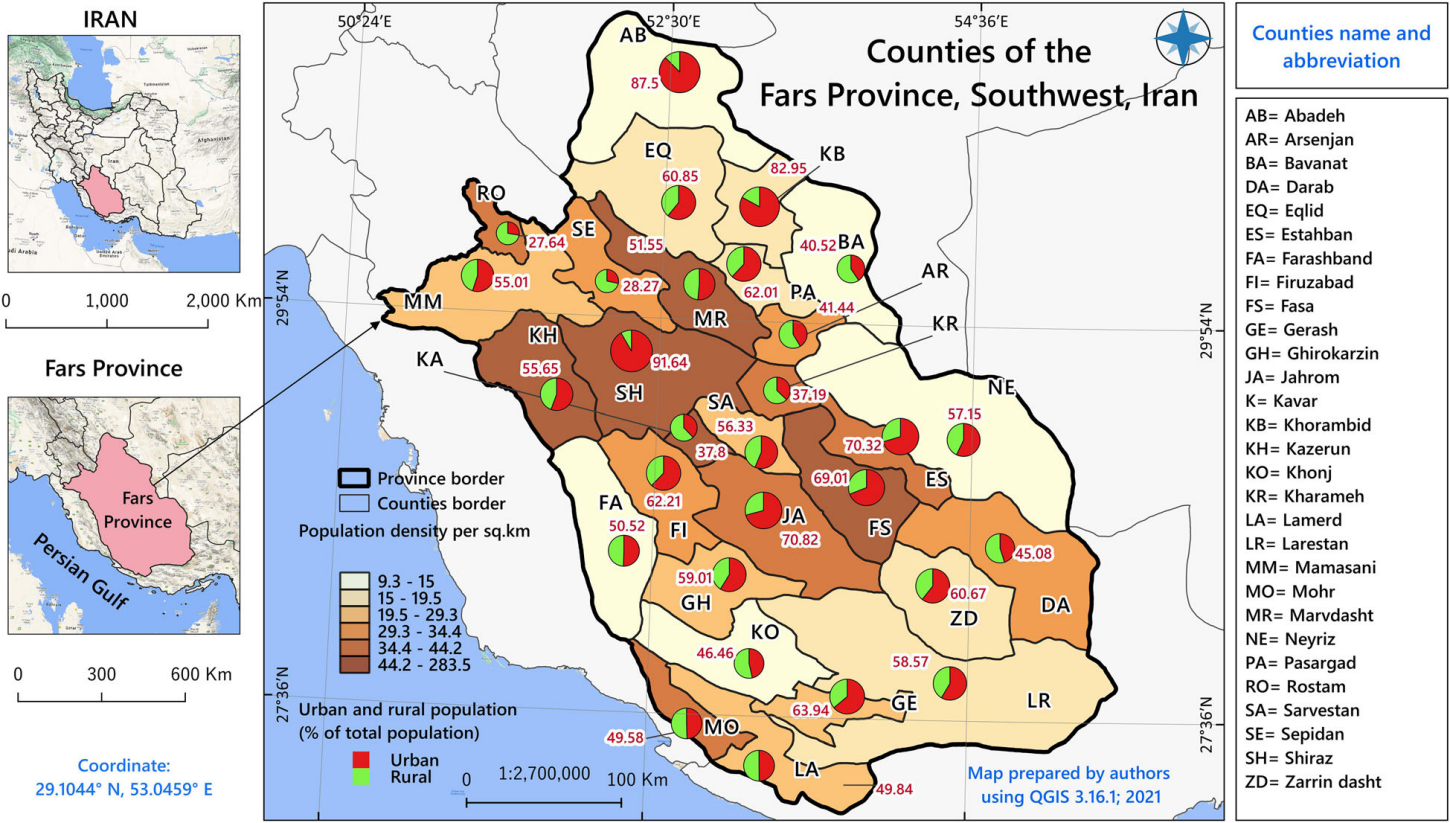
The geographic location and urbanization rate of each county in the study area

Fars Province is located in south-western Iran, and its capital is the city of Shiraz. It extends about 122,608 km^2^, comprising 29 counties with a total population of 4,851,274 residents, according to the census results released by the statistical center of Iran in 2016 [19]. Figure 1 illustrates the study area. Fars Province is located in a geographical region where the average annual sunshine time is very high (9–9.4 h per day) [20]. According to the Fars Meteorological Organization reports, the long-term average temperature throughout the province is reported between 11 and 23 °C. In some parts of the province, throughout most of the summer, the temperature rises to 50 °C. Additionally, this area receives a large amount of UV radiation over the summer, with higher amounts of reported radiation in regions with high elevation [21].

### Data source

The data on 4302 skin cancer cases recorded by Fars Cancer Registry over 6 years (mid-March 2011-mid-March 2017) were used for statistical and spatial analysis. The cancer data were recorded according to the official Persian calendar where the year starts at the spring equinox, which corresponds to around 20 March in the Gregorian calendar used in the West. The variables included in the study were patients’ residence address, gender, age, report date, and final topographical code. The patients’ addresses were aggregated to the counties of the study area. The country conducts a population census every 5 years. In our study, the population census data were available for the years 2011 and 2016, but we used the averaged data for the other years.

### Separating sun-exposed and non-sun-exposed parts of the body

In this study, we sought to assess the relationship between skin exposure to sunshine in both genders and the probability of skin cancer occurrence. Therefore, skin cancer sites were categorized based on sunshine exposure presence on male and female genders. Since in Iran, females have special clothing, the sunshine exposure to some parts of their body would be limited. For instance, in the female population, due to covering hair in public, the topographical site of “skin of scalp and neck” was considered without sunshine exposure. Although females do not cover their hair in private places, it is not significant because the indoor indirect sun exposure has a little amount of UV [22]. N/A (Not Applicable) means there was/were no/many specific site (s) of cancer on the body. Table 1 categorizes the topographical codes of skin cancer incidence along with their counts. As the table reveals, after “skin of other and unspecified parts of face” with 1945 patients, the “skin of scalp and neck” with 511 patients had the highest frequency.

**Table 1:** The categorization of skin cancer sites based on sunshine exposure for each gender in the study area during the period of 2011–2017

Skin Cancer site	ICD-10-CM Code (Topography)	Gender-based Sun Exposure	Count of males	Count of females
Skin of other and unspecified parts of face	C44.3	M:1, F:1	*1071*	*874*
Skin of scalp and neck	C44.4	M:1, F:0	*389*	*122*
Skin, NOS	C44.9	N/A	*252*	*190*
Eyelid	C44.1	M: 1, F:1	*196*	*196*
External ear	C44.2	M: 1, F:0	*258*	*44*
Skin of lip, NOS	C44.0	M: 1, F:1	*130*	*82*
Skin of trunk	C44.5	M:0, F:0	*99*	*67*
Skin of lower limb and hip	C44.7	M:0, F:0	*93*	*63*
Skin of upper limb and shoulder	C44.6	M:1, F:0	*78*	*43*
Overlapping lesion of skin	C44.8	N/A	*36*	*19*
Total			*2602*	*1700*
Number of cases of skin cancer based on sunshine exposure in males			*2122*	
Number of cases of skin cancer based on non-sun-exposure in males			*192*	
Number of cases of skin cancer based on sunshine exposure in females			*1152*	
Number of cases of skin cancer based on non-sun-exposure in females			*339*	

*NOS* Not Otherwise Specified*, M* Male*, F* Female*, N/A* Not Applicable*,* 1: With sun exposure, 0: Without sun exposure

### Incidence rates

**Fig. 2:**
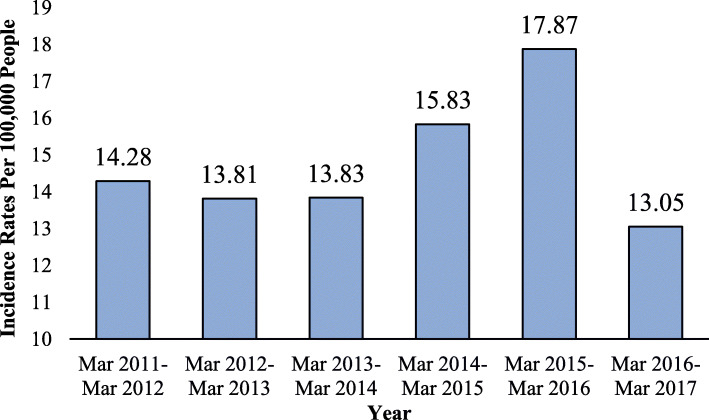
Skin cancer incidence rates per 100,000 people in the study area between 2011 and 2017

In this study, two different incidence rates were calculated. For descriptive statistics (Table 2 and Fig. 2), we calculated the age-specific incidence rates for each gender by dividing the number of skin cancer cases in each age group by the number of people in that age group. However, for spatial analyses and geographical mapping, the Empirical Bayesian Smoothing (EBS) approach was used to calculate the smoothed incidence rates to remove any potential bias regarding the counties’ population variability in the study area. The smoothed rate was expressed as a weighted average of the crude rate (r) and the prior estimate, say *θ*. The latter was estimated as a reference rate, typically the overall study area average or some other standard. By applying the local EBS technique, small units (i.e. those with a small population at risk) tend to have their rates adjusted considerably, whereas the rates would barely change for larger spatial units. As a result, the EBS estimation for the risk in spatial unit *i* was calculated by Eqs. 1–3 provided in the supplementary file 1. While easy to calculate, the estimate for the variance can yield negative values. In such instances, the conventional approach was to set *σ*^2^ to zero. As a result, the weight *w*_*i*_ becomes zero, which in essence equates the smoothed rate estimate to the reference rate [23].

**Table 2:** Skin cancer cumulative incidence rates per 100,000 people in different age groups by gender during 2011 to 2017

Age group	Male	Female	Total
< 30	3.76	3.68	3.72
30–39	23.26	21.25	22.27
40–49	62.72	61.57	62.15
50–59	205.57	145.01	175.67
60–69	491.08	298.70	391.44
70–79	1091.90	598.63	842.03
80–89	1352.75	816.95	1103.27
> = 90	1193.11	715.68	952.17
Total	105.72	71.13	88.51

#### Spatial cluster analysis

We used the EBS incidence rate instead of the crude incidence rate for spatial cluster analysis. Also, cluster and outlier analyses were done using the Anselin’s Local Moran’s Index (ALMI) [24]. There are two important types of spatial statistics to identify geographical variations; global cluster statistics and local spatial statistics. The global methods are more sensitive to departures from the null hypothesis (random distribution) but do not tell where the clusters are, which is possible when applying local methods [25]. We used the ALMI method since it is generally more accurate concerning measuring autocorrelation than other spatial statistics [24, 26]. This test calculates the *p*-value and z-score to illustrate and compare the apparent similarity. When the target area is surrounded by regions with identical rates of skin cancer, Low-Low (LL: low-risk area of cancer incidence) and High-High (HH: high-risk area of cancer incidence) concepts are used (positive correlation). High-Low (HL) and Low-High (LH) areas illustrate the dissimilarities and outliers of skin cancer incidence (negative correlation) [27].

#### Spatial hotspot analysis

We used Getis-Ord Gi* statistic for hotspot analysis. This measure examines spatial association at a local scale by comparing the sum of all feature values and the local sum of the values for the relevant features and the importance of its surrounding features [28].

#### Software

QGIS 3.16.1, a cross-platform and open-source desktop GIS application [29], was used to create the study area map. Microsoft Excel 2016 and IBM SPSS Statistics 20 were utilized for data preprocessing, descriptive analysis, and chart creation. ArcGIS 10.8 (ESRI, Redlands, CA, USA) and GeoDa 1.18 were used for spatial analysis. GeoDa is a free and open-source software tool for modeling and developing spatial patterns [30]. It was used for calculating the EBS rates.

## Results

### Descriptive results

The cumulative cancer incidence rate was 88.51 per 100,000 people during the study period (mid-march 2011 to mid-march 2017). Figure 2 shows skin cancer incidence rates in different years of the study period. The incidence rates had an increasing trend from 14.28 per 100,000 people in 2011 to 17.87 per 100,000 people in 2016, however, it was decreased to 13.05 per 100,000 people in 2017. Of 4302 patients with skin cancer, 2602 cases (60%) were male. Males with a value of 105.71 per 100,000 had a higher cumulative cancer incidence rate than females (71.12 per 100,000). Table 2 illustrates the cumulative incidence rates in different age groups. According to this table, the rate of reported skin cancer cases in the 80–89 age group (in both male and female genders) was higher compared to other age groups. The geographical distribution of rates is provided in the spatial findings section.

The cancer cumulative incidence rate in patients who were not exposed to sunshine was 10.95 per 100,000. This rate increased to 67.49 per 100,000 in patients who were exposed to the sun. The rate in patients not exposed to the sun was 7.80 per 100,000 in the male group and 14.18 per 100,000 in the female group. However, the cumulative incidence rates in the sunshine-exposed group increased to 86.22 per 100,000 in the male group and 48.20 per 100,000 in the female group.

### Spatial results

#### Rate distribution

**Fig. 3:**
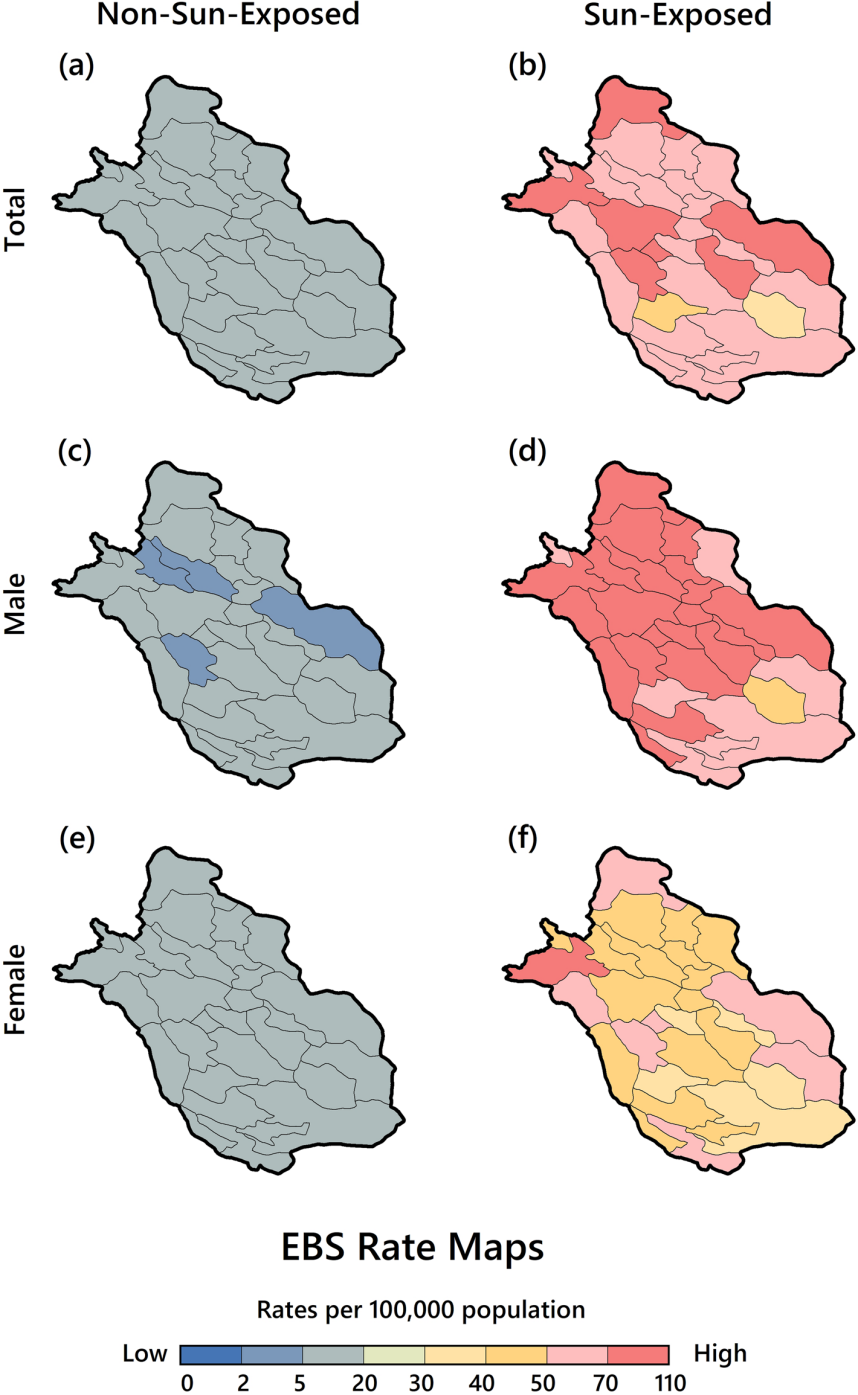
EBS rates of skin cancer in Fars Province based on gender and sunshine exposure of skin sites

Figure 3 shows the maps of skin cancer EBS incidence rates by gender and category (SE: Sunshine-Exposed or NSE: Non-Sunshine-Exposed). According to these maps, in an NSE status, the incidence rates in the whole study area were below 20 per 100,000 (Fig. 3a) and represented a homogeneous geographical distribution pattern. As Fig. 3c and e illustrate, this homogeneous geographical pattern of low rates is repeated in male and female groups. The difference is that females’ rate (13.89 per 100,000) was higher than males’ rate (7.15 per 100,000).

When patients were exposed to sunshine, a different pattern was observed. Some counties experienced very high rates (more than 70 per 100,000) (Fig. 3b) (including Mamasani, Abadeh, Kavar, Neyriz, Shiraz, Fasa and Firuzabad). In the male group (Fig. 3d), nearly 80% of counties experienced these high rates. In the female group (Fig. 3f), high rates are also seen in all counties, although only one county (Mamasani) had the highest rate (70.06 per 100,000).

The result highlights a significant difference in the geographical distribution of EBS rates between the two categories, and in the male group, all counties experienced higher EBS incidence rates. Specific rates for each county and category are available via supplementary file 2.

### Cluster analysis

#### Clusters/outliers

**Fig. 4:**
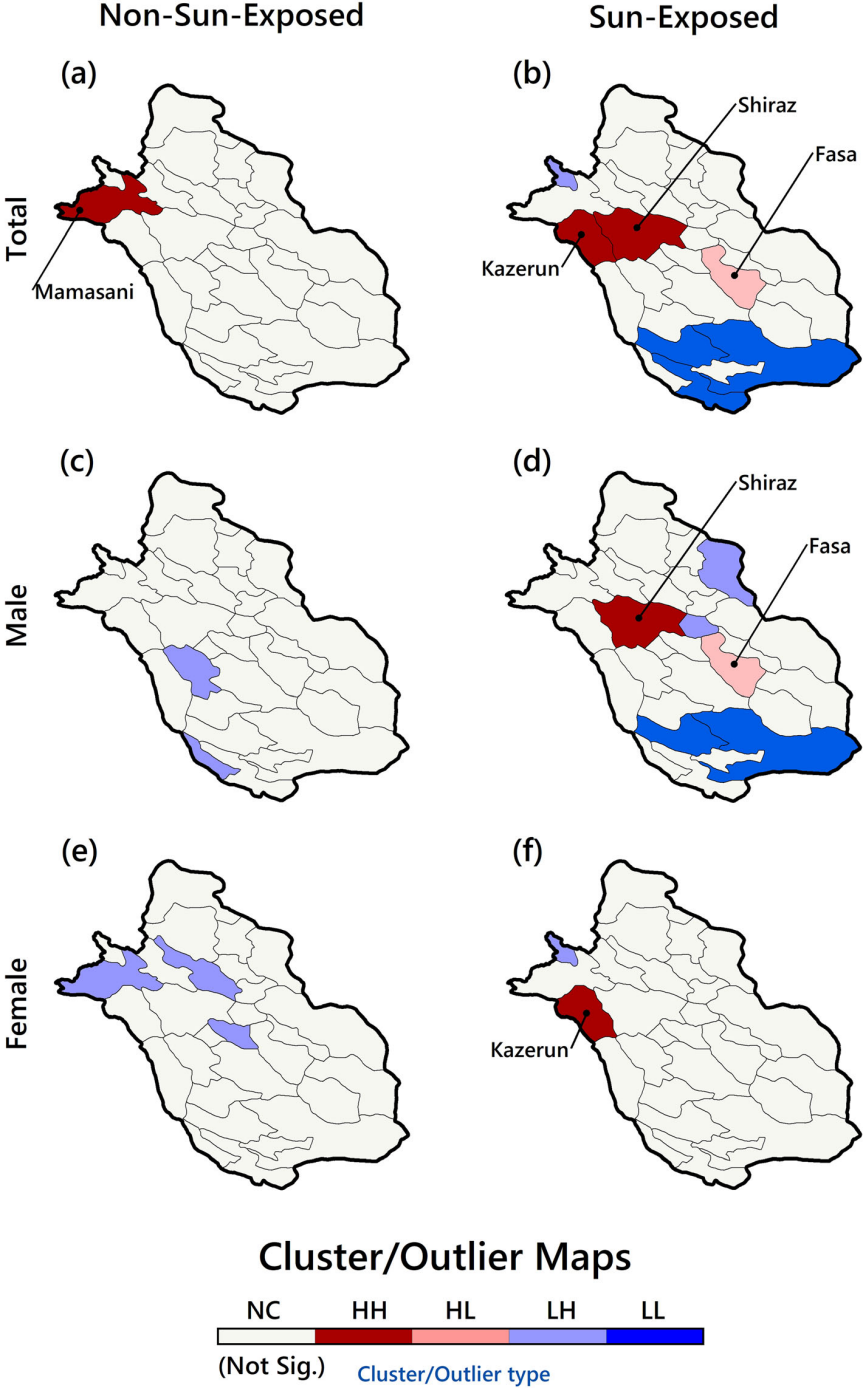
EBS Cluster maps of skin cancer in Fars Province based on gender and sunshine exposure of skin sites

The ALMI related maps are depicted in Fig. 4. As established, based on total incidence EBS rates in NSE cases (Fig. 4a), one HH cluster was recognized, which included the northwest county. That is, the rates in this region and its neighbours were higher compared to the rest regions. Again, in the same NSE category and the male group, two outliers (LH) were formed (Fig. 4c); these LH clusters highlight that the counties themselves had a very low rate, but the neighbours had high rates. In the female group (Fig. 4e), in the NSE category, three outliers (LH) were formed.

In the SE category, in terms of total EBS rates (Fig. 4b), two HH clusters in the west, one LH outlier in the northwest, one HL outlier in the centre, and three LL clusters in the south were formed. The pattern of the male group (Fig. 4d) almost follows the total EBS rates clustering pattern. The difference is that the number of LH outliers has increased in the north-eastern regions. In the female group (Fig. 4f), only one HH cluster and one LH outlier were formed in the northwest.

In summary, there is a diversity of spatial clusters and outliers in total EBS rates maps. The clustering pattern of the male group followed the pattern of total clustering. But in the female group, this clustering was significantly different and strongly random.

#### Hot spot analysis

**Fig. 5:**
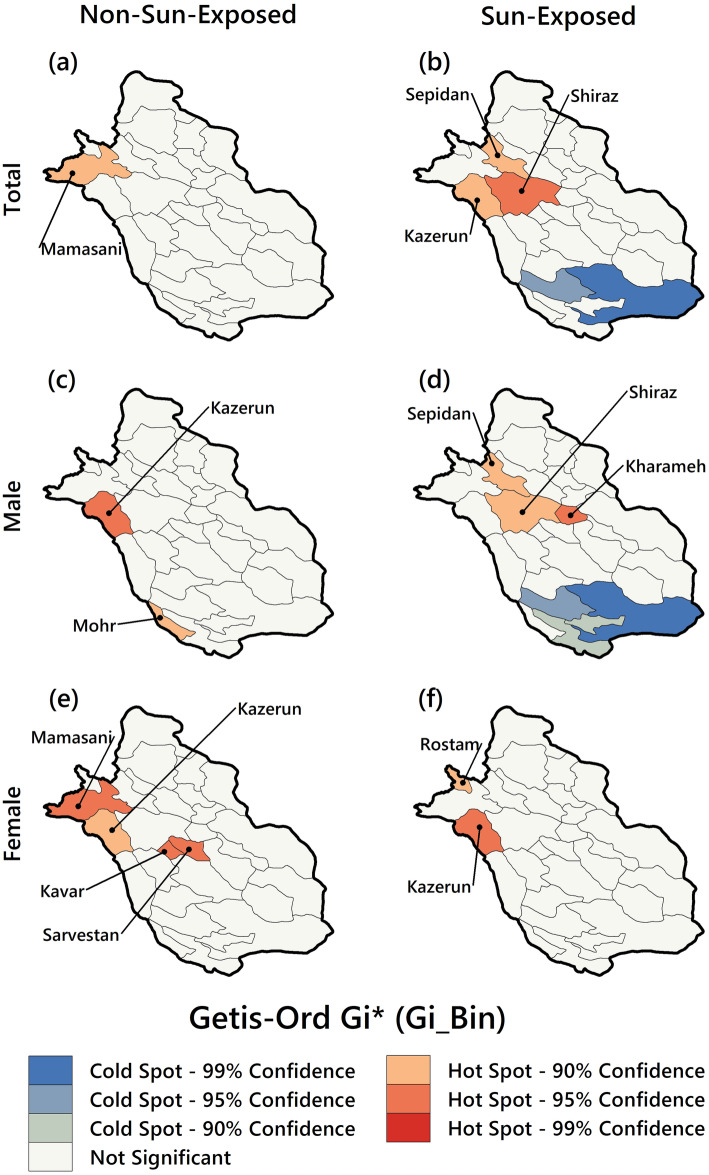
Hot spot and cold spots of skin cancer incidence in Fars province from mid-March 2011 to mid-March 2017

Figure 5 shows the results of Getis-Ord Gi* statistic for cancer EBS rates in two categories (NSE and SE) for the study population (Total, male, and female). Figure 5a illustrates the total skin cancer EBS incidence hotspot map in the NSE category. According to this map, only one county in the northwest (Mamasani) was identified as a hotspot with higher Z-scores, lower *p*-values, and with 90% confidence. In this category and male group (Fig. 5c), two hotspots were identified in the northwest (Kazerun) and southwest (Mohr) with 90 and 95% confidence. In the female group (Fig. 5e), four hotspots were formed in the study area’s central and west parts with 95% confidence (Sarvestan, Kavar, Kazerun, and Mamasani).

Figure 5b depicts the total skin cancer incidence EBS hotspot map in the SE category. Unlike the NSE group, in this category, three different counties (Shiraz, Kazerun, and Sepidan) were identified as hotspots with 90–95% confidence, which were all concentrated in the north-western area. In other words, the pattern of hotspots was different in the two categories. In the SE category and the male group (Fig. 5d), three hotspots, Shiraz in the west, Kharameh in the center and Sepidan in the north-west were formed with 90 and 95% confidence respectively. According to Fig. 5f, the pattern of hotspots in the female group was different. In this group, two hotspots (Rostam in the northwest and Kazerun in the west) were formed in the study area with 90 and 95% confidence, respectively.

As a result, hotspots were formed mostly in the west and northwest. Also, the pattern of hotspots in the female group was different from the male group. Some counties (Mamasani) in the NSE category and some others (Shiraz and Kazerun) in the SE category were the most frequent hotspots.

## Discussion

Our descriptive results demonstrate that the incidence of skin cancer has increased during the first 5 years (2011–2016), but decreased in 2017. In addition, the findings also show that the incidence of skin cancer was higher in males. Similar to our results, in the study of Razi et al. in Iran in 2008, it was claimed that the incidence rate was higher in male gender compared to female, 27.31 and 19.16, respectively [31]. The cumulative incidence of skin cancer in Fars Province was 88.51 per 100,000 in the period of 2011 to 2017. Further, the annual skin cancer incidence rate during the study period has been shown in Fig. 2. Previous research has also pointed to the high incidence of skin cancer in this province across the country [5, 16]. According to a study in Iran in 2009, Semnan and Isfahan provinces had the highest incidence rate of skin cancer among males with 34.9 and 30.8 per 100,000 in the country. Furthermore, in terms of females’ skin cancer, Semnan and Yazd provinces had the highest incidence rate with 26.1 and 24.1 per 100,000, respectively [5]. Our results also show that most people with skin cancer were elderlies (age > 70). These findings are in accordance with previous research results [5, 10, 14, 16]. According to official reports, the study area has a high ageing ratio (about 10% of the population are 60 years or older). However, this did not lead to bias in our results because we used the age-specific cancer rate to compare age groups’ incidence rate [32]. We found out that when skin areas were exposed to sunshine, skin cancer smoothed incidence rates rise sharply (10.95 per 100,000 in NSE to 67.49 per 100,000 in SE cases). This increase was more in men (from 7.80 to 86.22) than women (from 14.18 to 48.20) (supplementary file 2).

There are three key findings related to our spatial analysis that are further discussed and compared with related research. The first finding is that a different geographical distribution pattern of cancer incidence rates was formed between two categories (NSE and SE) and between males and females in the study area. This finding is consistent with the previous literature [33]. In the first category, NSE, the distribution was homogeneous, and in the second category (SE), the distribution was heterogeneous. For example, according to our findings, Shiraz is a county with higher male cancer rates, as reported in another previous study [5]. Others have confirmed the association between prolonged sunshine exposure and skin cancer [5, 16]. This finding is also consistent with the study of Cecconi et al. [10], which concluded that the high rate of skin cancer in men and its association with intense sunlight exposure is quite consistent. They noted that men in Italy’s coastal areas are subject to extreme sunshine exposure to take a sunbath. But in Iran and in Fars Province, most parts of the face, neck and hands are unintentionally exposed to the sun as a result of daily activities. This finding may be explained by the idea that, in the whole study area, men are more likely to engage in outdoor social and economic activities than women. Furthermore, the urbanization rate among the counties (Fig. 1) showed that most of the counties identifying as skin cancer high-risk areas had lower rates of urbanization. This might be because in rural areas people work more outdoors and are exposed more to sunlight. Our results also show that although the whole studied area is affected by prolonged sunshine exposure, some areas have experienced much higher cancer rates (especially in the male group), which could be due to various reasons including Ozone depletion, latitude, altitude, weather conditions, environmental pollutants, chemical carcinogens, consumption of drinking water containing inorganic arsenic, skin color, and smoking [34].

The second finding (the ALMI results) shows a clear difference between the two groups (NSE and SE) and between the male and female groups in terms of spatial clustering. In the first category, a small number of HH clusters was formed, but in the second group, the number of HH clusters was higher and closely resembled a clustered pattern. Geographically, HH clusters were formed in the western and north-western regions, and LL clusters were often created in the southern and southeastern parts. There seem to be common factors in the north-western areas that have had a more significant impact on skin cancer incidence in some counties. This study showed that the incidence of cancer in men had a higher effect on the spatial clustering pattern. Previous studies [5, 11, 14, 35] have noted an association between the harmful sunshine exposure (e.g., UV) and high skin cancer rates in some areas and the formation of HH clusters in more populated areas (such as Shiraz in this case). As Augustin et al. [36] have pointed out, in addition to factors affecting the incidence of skin cancer, such as harmful sun rays, there are essential geographical factors (such as air pollution, elevation, and wind blow intensity) and socio-economic factors (the type of employment, population density, and age) that are involved in the formation of HH areas (high risk); This could be because of the fact that skin cancer is more common in men who work outdoors and have to stay exposed to sunshine for extended periods [37].

The third main finding (the results of Getis-Ord Gi* statistic) is the identification of hot spots in the study area based on EBS rates in two categories (NSE and SE) for the study population (Total, male and female). Hotspots are formed mostly in the west and northwest in two categories (NSE and SE). The hotspots pattern was different in the female and male groups, but there was a hotspot in both groups. Some counties in the NSE category and some others in the SE category were the most frequent hotspots. In the female group, three hot spots were formed in the NSE category. It seems to be due to individual, environmental, or social factors other than sunshine exposure. As mentioned above, some studies concluded that skin cancer is more common in men who work outdoors [37]. Whereas other researchers have found hotspots [5, 12, 13, 15], the present study has identified two hotspots as high-risk areas (Shiraz and Kazerun), which can be prioritized for preventive or curative measures.

### Recommendations

Skin cancer seems to be a severe challenge in the coming years for areas where sunshine radiation’s duration and intensity are high. Sustainable training for jobs that require prolonged exposure to sunshine is an essential step in preventing skin cancer in this group. Educating children from an early age to protect their skin against harmful sunshine radiation is an issue that needs to be addressed by education and healthcare policymakers. In regions where the duration and intensity of radiation are high, educational programs for promoting preventive tools are essential. Various other genetic, individual, and socio-economic factors are also involved in the incidence of skin cancer. Therefore, it would be useful to extend the current findings by studying the association between environmental variables such as sunshine duration and intensity, altitude, temperature, and air pollution using accurate data and patients’ exact geographical location (point features data).

### Limitations

The present study’s limitations naturally include lack of access to accurate spatial data for skin cancer patients (point address) and accurately recorded data on UV radiation, sunshine duration, and intensity on a local scale. There were only 192 not sun-exposed males in the study area. Splitting this number over the 29 counties can affect our spatial analysis. Despite these limitations, the present study has enhanced our understanding of the relationship between spatial patterns of skin cancer incidence rates (in two sex groups) and NSE/SE categories.

## Conclusions

We identified the fact that the incidence of skin cancer increased from 2011 to 2016 but then decreased in 2017. Patients exposed to the sunshine, especially in the male group, experienced much higher incidence rates than those who were not exposed to the sun. With a heterogeneous spatial pattern, hotspots were identified in NSE and SE categories in the study area. It can be concluded that, in terms of total skin cancer incidence rates, hotspots were formed in and around densely populated areas. Researchers and policymakers can significantly benefit from the spatial analyses of skin cancer incidence. These analyses can provide useful and timely prevention policies as well as highly-considered monitoring techniques in high-risk regions.

## Supplementary Information

**Additional file 1.**

**Additional file 2.**

## Data Availability

The datasets are available from the corresponding author on reasonable request.

## Abbreviations

MM: Malignant Melanoma
NMSC: Non-Melanoma Skin Cancer
BCC: Basal Cell Carcinoma
SCC: Squamous Cell Carcinoma
ASR: Age-Standardized Incidence Rate
UV: Ultraviolet
GIS: Geographic Information Systems
N/A: Not Applicable
NOS: Not Otherwise Specified
EBS: Empirical Bayesian Smoothed
ALMI: Anselin’s Local Moran’s Index
LL: Low-Low
HH: High-High
HL: High-Low
LH: Low-High
API: Application Programming Interface
OSM: OpenStreetMap
NSE: Non-sunexposure/exposed
SE: Sunexposure/exposed
SDP: Spatial Distribution Pattern

## References

[1] Razi S, Rafiemanesh H, Ghoncheh M, Khani Y, Salehiniya H (2015). Changing trends of types of skin Cancer in Iran. Asian Pac J Cancer Prev.

[2] Apalla Z, Nashan D, Weller RB, Castellsague X (2017). Skin Cancer: epidemiology, disease burden, pathophysiology, diagnosis, and therapeutic approaches. Dermatol Ther.

[3] Afzali M. Epidemiology of skin cancer and changes in its trends in Iran. J Kashan Univ Med Sci. 2013;7:501–11 URL: http://feyz.kaums.ac.ir/article-1-2042-en.html. Accessed 20 Nov 2021.

[4] Qureshi A, Laden F, Colditz G, Hunter D (2008). Geographic variation and risk of skin Cancer in US WomenDifferences between melanoma, squamous cell carcinoma, and basal cell carcinoma. Arch Intern Med.

[5] Pakzad R, Ghoncheh M, Pournamdar Z, Pakzad I, Momenimovahed Z, Salehiniya H, Towhidi F, Makhsosi BR (2016). Spatial analysis of skin Cancer incidence in Iran. Asian Pac J Cancer Prev.

[6] Fartasch M, Diepgen T, Schmitt J, Drexler H (2012). The relationship between occupational sun exposure and non-melanoma skin cancer: clinical basics, epidemiology, occupational disease evaluation, and prevention. Dtsch Arztebl Int.

[7] Ananthaswamy HN (2001). Sunlight and skin Cancer. J Biomed Biotechnol.

[8] Sahar L, Foster SL, Sherman RL, Henry KA, Goldberg DW, Stinchcomb DG, Bauer JE (2019). GIScience and cancer: state of the art and trends for cancer surveillance and epidemiology. Cancer..

[9] Hashtarkhani S, Tabatabaei-Jafari H, Kiani B, Furst M, Salvador-Carulla L, Bagheri N (2021). Use of geographical information systems in multiple sclerosis research: a systematic scoping review. Mult Scler Relat Disord.

[10] Cecconi L, Busolin A, Barbone F, Serraino D, Chiarugi A, Biggeri A (2016). Spatial analysis of incidence of cutaneous melanoma in the Friuli Venezia Giulia region in the period 1995–2005. Geospat Health.

[11] Amancio CT, Nascimento LF (2014). Cutaneous melanoma in the state of Sao Paulo: a spatial approach. An Bras Dermatol.

[12] Ferreira FR, Nascimento LF (2016). Mortality due to cutaneous melanoma in south region of Brazil: a spatial approach. An Bras Dermatol.

[13] Hu S, Sherman R, Arheart K, Kirsner RS (2014). Predictors of neighborhood risk for late-stage melanoma: addressing disparities through spatial analysis and area-based measures. J Invest Dermatol.

[14] Ray GT, Kulldorff M, Asgari MM (2016). Geographic clusters of basal cell carcinoma in a northern California health plan population. JAMA Dermatol.

[15] Chang N-B, Feng R, Gao Z, Gao W (2010). Skin cancer incidence is highly associated with ultraviolet-B radiation history. Int J Hyg Environ Health.

[16] Zayeri F, Kavousi A, Najafimehr H (2015). Spatial analysis of relative risks for skin cancer morbidity and mortality in Iran, 2008-2010. Asian Pac J Cancer Prev.

[17] Mohebbipour A, Alipour S, Ahari SS, Amani F, Farzaneh E. Investigating the geographical distribution of skin cancer (BCC type) in Ardabil province via GIS. Int J Res Med Sci. 2015;3(8). 10.18203/2320-6012.ijrms20150332.

[18] Ghoncheh M, Koohi F, Salehiniya H (2015). Epidemiology and Trend of Skin Cancer Incidence in Southern Iran. J Dermatol Cosmetic.

[19] Statistical Centre of Iran (SCI). Population and Housing Censuses of 2016: Statistical Center of Iran; 2018 [cited 2020, November 21 ]. Available from: https://www.amar.org.ir/english/Population-and-Housing-Censuses

[20] Ahmadi H, Ahmadi F (2019). Evaluation of sunshine duration and temporal–spatial distribution based on geostatistical methods in Iran. Int J Environ Sci Technol.

[21] Machhi J, Herskovitz J, Senan AM, Dutta D, Nath B, Oleynikov MD, Blomberg WR, Meigs DD, Hasan M, Patel M, Kline P, Chang RCC, Chang L, Gendelman HE, Kevadiya BD (2020). The natural history, pathobiology, and clinical manifestations of SARS-CoV-2 infections. J Neuroimmune Pharmacol.

[22] Saric-Bosanac SS, Clark AK, Nguyen V, Pan A, Chang FY, Li CS, et al. Quantification of ultraviolet (UV) radiation in the shade and in direct sunlight. Dermatol Online J. 2019;25(7). 10.5070/D3257044801. 31450273

[23] Anselin L, Lozano N, Koschinsky J (2006). Rate transformations and smoothing. Urbana.

[24] Anselin L (1995). Local indicators of spatial association—LISA. Geogr Anal.

[25] Kiani B, Raouf Rahmati A, Bergquist R, Hashtarkhani S, Firouraghi N, Bagheri N, Moghaddas E, Mohammadi A (2021). Spatio-temporal epidemiology of the tuberculosis incidence rate in Iran 2008 to 2018. BMC Public Health.

[26] Anselin L, Syabri I, Kho Y. GeoDa: an introduction to spatial data analysis. Handbook of applied spatial analysis: Springer; 2010. p. 73–89. DOI:10.1007/978-3-642-03647-7_5.

[27] Bagheri N, Furuya-Kanamori L, Doi SAR, Clements ACA, Sedrakyan A (2017). Geographical outcome disparities in infection occurrence after colorectal surgery: An analysis of 58,096 colorectal surgical procedures. Int J Surgery (London, England).

[28] Kim SM, Choi Y. Assessing Statistically Significant Heavy-Metal Concentrations in Abandoned Mine Areas via Hot Spot Analysis of Portable XRF Data. Int J Environ Res Public Health. 2017;14(6). 10.3390/ijerph14060654. 10.3390/ijerph14060654 PMC5486340 28629168

[29] QGIS: A Free and Open Source Geographic Information System 2020 [cited 2020, December 1 ]. Available from: https://qgis.org/en/site/

[30] GeoDa: An Introduction to Spatial Data Analysis 2020 [cited 2020, December 1]. Available from: https://geodacenter.github.io/

[31] Razi S, Enayatrad M, Mohammadian-Hafshejani A, Salehiniya H, Fathali-Loy-Dizaji M, Soltani S (2015). The epidemiology of skin Cancer and its trend in Iran. Int J Prev Med.

[32] Ministry of Cooperatives Labour and Social Welfare (MCLSW). Study of aging indicators and forecasting the aging trend in Iran until 2040. Ministry of Cooperative Labour and Social Welfare (MCLSW) 2015;http://www.amarkar.ir/handler/getfile.ashx?type=pub&id=320. Accessed 15 Nov 2021.

[33] Rohani-Rasaf M, Abdollahi M, Jazayeri S, Kalantari N, Asadi-Lari M (2013). Correlation of cancer incidence with diet, smoking and socio-economic position across 22 districts of Tehran in 2008. Asian Pac J Cancer Prev.

[34] Fabbrocini G, Triassi M, Mauriello MC, Torre G, Annunziata MC, De Vita V (2010). Epidemiology of skin cancer: role of some environmental factors. Cancers (Basel).

[35] McKinley JM, Ofterdinger U, Young M, Barsby A, Gavin A (2013). Investigating local relationships between trace elements in soils and cancer data. Spatial Statistics.

[36] Augustin J, Kis A, Sorbe C, Schäfer I, Augustin M (2018). Epidemiology of skin cancer in the German population: impact of socioeconomic and geographic factors. J Eur Acad Dermatol Venereol.

[37] Mackie R, Quinn A. Non-melanoma skin cancer and other epidermal skin tumours. Rooks Textbook Dermatol. 2004:1801–50. 10.1002/9780470750520.ch36.

