# Supplementary material for: Spatial epidemiology of skin cancer in Iran: separating sun-exposed and non-sun-exposed parts of the body
Source: Arch Public Health. 2022 Jan 20;80:35. doi: 10.1186/s13690-022-00798-2 (PMC8772111; doi:10.1186/s13690-022-00798-2)
Supplement: Supplementary file 1 — Additional file 1. [file 13690_2022_798_MOESM1_ESM.docx]

| $\pi_{i}^{EB}=w_{i}r_{i}+(1-w_{i})\theta$ | Eq.1 |
| --- | --- |
| $\pi_{i}^{EB}$is the skin cancer empirical Bayesian smoothed rate for geographical unit i. $r_{i}$ is the skin cancer crude rate in area i, *θ* is the skin cancer prior distribution (reference rate), and $w_{i}$ is the spatial weight that is calculated by equation 2. |  |
| $w_{i}=\frac{\sigma^{2}}{(\sigma^{2}+\mu/P_{i})},$ | Eq.2 |
| $P_{i}$is the population at risk in area *i*, $\mu$ and $\sigma^{2}$ are the mean and variance of the skin cancer prior distribution. In the empirical Bayes approach, the mean $\mu$ and variance $\sigma^{2}$ of the prior (which determine the scale and shape parameters of the Gamma distribution) are estimated from the data. For $\mu$ this estimation is simply the reference rate (the same reference used in the computation of the standardized or relative risk). |  |
| $\sigma^{2}=\frac{\sum_{i=1}^{i=n} P_{i}(r_{i}-\mu)^{2}}{\sum_{i=1}^{i=n} P_{i}}-\frac{\mu}{\sum_{i=1}^{i=n} P_{i}/n}.$ | Eq.3 |
| $\sigma^{2}$is the variance of the skin cancer prior distribution. $P_{i}$is the population at risk in area *i*, $\mu$is the mean of the skin cancer prior distribution, $r_{i}$ is the skin cancer crude rate in area i, and n is the number of spatial features (counties). |  |
